# Direct Differentiation of Human Primary Fibroblast into Hematopoietic-Like Stem Cells; A New Way without Viral Transduction

**DOI:** 10.22074/cellj.2020.6846

**Published:** 2020-09-08

**Authors:** Sina Habibi, Gholamreza Khamisipour, Narges Obeidi, Saeedeh Zare Jaliseh

**Affiliations:** 1 Department of Hematology, Faculty of Allied Medicine, Bushehr University of Medical Sciences, Bushehr, Iran; 2 Department of Anatomy, Faculty of Medical Sciences, Tarbiat Modares University, Tehran, Iran

**Keywords:** Direct Differentiation, Electromagnetic Waves, Fibroblast, Hematopoietic Stem Cells, Reprogramming

## Abstract

**Objective:**

The aim of this study was to investigate the possibility of producing safe hematopoietic stem cells without the use of viral infectious agents that can be used in stem cell transplantation.

**Materials and Methods:**

In this experimental study, after single layer cell formation, human primary fibroblast cells were treated with static electromagnetic fields of 10 and 15 milli Tesla (mT) for 20 minutes each day for seven consecutive days. On the seventh day and immediately after the last radiation, the cells were added to the wells containing specific hematopoietic stem cell expansion media. After 21 days and colony formation, the cells belonging to each group were evaluated in terms of the expression of CD34, CD38, and GATA-1 genes using quantitative real-time polymerase chain reaction (PCR), as well as surface marker expression of CD34 by flow cytometry.

**Results:**

Exposure to 10 mT and 15 mT electromagnetic field increased the expression of CD34 and CD38 genes (P<0.05). This increase in gene expression levels were 2.85 and 1.84 folds, respectively, in the 10mT group and 6.36 and 3.81 folds, respectively, in the 15 mT group. The expression of the GATA-1 gene in the 10 mT and 15 mT groups was not significantly different from that of the control group (P<0.05). Electromagnetic waves caused a marked increase in the expression of the CD34 marker at the surface of reprogrammed cells. The rate of expression was about 42.3% in the 15 mT group and 23.1% in the 10 mT group.

**Conclusion:**

The presence of human primary fibroblasts exposed to electromagnetic fields can increase the expression of specific hematopoietic genes. This method can be suitable for reprogramming cells differentiated into hematopoietic- like stem cells and does not pose the risks of retroviral use.

## Introduction

Stem cells are primary cells that can differentiate into various cells, including embryonic stem cells (ES cell) and adult stem cells. These cells can divide and rebuild themselves and become specialized cells, so they can be used to cure different diseases, such as diabetes, arthritis, and spinal cord injury in the future (1).

In recent years, scientists have applied viral vectors containing sex-determining region Y (*SOX2),* octamerbinding transcription factor 4 *(OCT4), *Kruppel-like factor 4 *(KLF4)* and Avian myelocytomatosis virus oncogene cellular homolog (*c-MYC)* genes in somatic cells to produce cells similar to embryonic cells that are able to form cells of all three embryonic layers. These cells called induced pluripotent stem cells (iPSCs) (2-4). Different types of somatic cells obtained from pluripotent stem cells could be used in regenerative medicine (5). Viruses that are used to transfer these genes to cells enter their genetic material (transgenes) into the genome of host cells (6). Transgenes are mainly silenced in iPS cells, but the reactivation of such transgenes (primarily the transgene encoding *c-MYC*) could lead to tumorigenesis (7). These genes can be dangerous, so the clinical use of iPS cells is currently impossible (8, 9).

The iPS cell potentially could overcome two crucial barriers related to human ES cells: immune rejection after transplantation and ethical concerns about the use of human embryos (7, 9).

Michael Faraday first introduced electromagnetic induction. This theory indicates that magnetic field fluctuations can create an electrical current in conductors that are close to it. Whether electromagnetic waves are constant or alternating in time, each one has physical characteristics that interfere with biological organisms [plants, animals, and humans (10)].

From the point of view, organisms are electromagnetic systems, and they use magnetism to emit proteins, interact in molecular systems of cell membrane, and disseminate information through nerve systems. The interest in interactions between the magnetic field and living organisms triggers a series of epidemiological studies. These studies suggest a weak correlation between exposure to magnetic fields and the incidence of various types of cancer (11).

The effects of a strong magnetic field on the metabolic activity of leukemia cells were investigated. In this experiment, human leukemic cells (HL- 60) were subjected to a 1T static magnetic field for 72 hours, resulting in a significant reduction in the metabolic activity of cancer cells (12). The magnetic field affects the non-sexual division of dictyostelium discoideum as a model for human disease. When the protozoa were subjected to the electromagnetic field at a frequency of 50 hertz (Hz) and the intensity of 300 microteslas (μT) for 24 hours, the net rate of nonsexual division of this protozoan was changed (13). *In vitro* studies performed on human cells show increased cell proliferation in immune cells and also promote new angiogenesis in endothelial cells exposed to electromagnetic waves (14).

Huangfu et al. (15) used early fibroblast cells to form iPSCs. In this study, only *OCT4* and *SOX2* genes were used for reprogramming using valproic acid. The results showed that it is possible to reprogramming cells by means of the pure chemicals, and more secure methods can be used for the generation of iPSC. In another study conducted by Baek et al. (16) on the reprogramming of somatic cells, they employed both *OCT4* and *SOX2* genes and the electromagnetic waves, iPSC-like generations were formed in the culture media. It was also observed that electromagnetic waves could be used well instead of using *c-MYC* and *KLF4* genes.

In this study, human fibroblast cells were exposed to static electromagnetic fields, and after transferring to a differentiated media, the production of hematopoietic stem cells in these cells were induced and tested.

## Materials and Methods

This study is experimental research approved as a thesis with an Ethical code number of "IR.BPUMS. REC.1395.201" in Bushehr University of Medical Sciences.

Experiments were carried out in two separate groups, which were exposed to static electromagnetic fields under the radiation of 10 and 15 milli Tesla (mT). Each experiment was performed triplicate, and the entire experiment was repeated at three different times.

### Electromagnetic field exposure

After single-cell layer formation, cells were exposed to static electromagnetic fields of 10 and 15 mT for 20 minutes each day for seven consecutive days. A device designed and built in the lab produced the electromagnetic field.

### Structural design of the electromagnetic field generator device

The generator used to stimulate the cells has been manufactured and calibrated by the Persian Gulf University, Bushehr, Iran. Briefly, three-column coils, 12 cm in diameter and made of 2300 turns of enamel copper wire (0.6 mm in diameter), was mounted in a horizontal arrangement. Two ends of these wires can be connected to a suitable voltage. This device was designed to produce a static electromagnetic field with an energy range between 0 and 320 V and a magnetic field strength range between 0 and 40 mT. A line power supply powered the entire apparatus and connected directly to the variac. The electricity was converted into DC through an ACDC rectifier. After adjusting the intensity of the field with gaussmeter, a 4-well culture plate was placed at the center of a uniform field area. Temperature near the culture plates was monitored, and no variation was recorded throughout the experiments. The intensity of the electromagnetic field used in this protocol was 10 to 15 mT, and the application time was 20 minutes.

### Cell culture

Human primary fibroblasts HU02 (Stem Cell Technology, bonbiotech, Iran) were cultured in 4-well plates containing Dulbecco’s Modified Eagle’s Medium with Ham’s F-12 Nutrient Mixture (DMEM.F12 Medium, Caisson, USA), which included 100 IU/mL penicillin, 100 μg/ml streptomycin in 0.9% saline (Caisson, USA), and 10 % fetal bovine serum (FBS, Gibco, USA), 1% non-essential Amino acid solution (Sigma, USA) and incubated at 37°C at 5 % CO_2_. After 24 hours and singlecell layer formation, non-adherent cells were eliminated by medium exchange, and the plate was exposed to electromagnetic radiation for seven days.

On the seventh day and immediately after the last radiation, the cells were separated from the well with 0.25% trypsin-EDTA (Caisson, USA) enzyme solution. Following the centrifugation of cells, they were transferred to a new 4 well-plate (10,000 cells per well) containing Stem MACS HSC Expansion Media (Miltenyi Biotec, Germany) supplemented with Stem MACS HSC Expansion Cocktail (Miltenyi Biotec, Germany) containing the human recombinant growth factor Flt3- Ligand, stem cell factor (SCF) and thrombopoietin (TPO). After 21 days of proliferation and colony formation, cells were examined for the expression of the specific hematopoietic genes [*CD34, CD38,* and GATA-binding factor 1 (*GATA-1*)] by quantitative real-time polymerase chain reaction (qRT-PCR) and hematopoietic surface marker (CD34) by flow cytometry.

### The examined genes

The *CD34, CD38, *and *GATA-1* genes were studied as target genes. For the determination of the relative gene expression levels, the hypoxanthine-guanine phosphoribosyl transferase (*HPRT*) gene was used as a reference gene.

The sequences of *CD34, CD38, GATA-1*, and* HPRT* genes were extracted from the ncbi.nlm.gov, and related primers were designed using the Gene Runner software version 6.5. After designing, using the NCBI site tool, the primer sequence (Table 1) was blasted with the entire human genome, and the primer properties for complete complementary areas were fully assured.

### Quantitative real-time polymerase chain reaction

For the quantitative real-time PCR (qRT-PCR) analysis, RNA was isolated using a YTzol Pure RNA (Yekta Tajhiz Azma, Iran) according to the manufacturer’s protocol. Complementary DNA was produced with the cDNA Synthesis kit (Yekta Tajhiz Azma, Iran) according to the manufacturer’s protocol. qRT-PCR reactions were set up in triplicate with the Amplicon RealQ Plus 2x Master Mix Green (Amplicon, Denmark) and run on a Step-One plus real-time PCR system (Applied Biosystems, USA) according to the manufacturer’s protocol. Gene expression data were reported as relative expression to *HPRT*. qRTPCR products were checked by gel electrophoresis according to the product sizes.

### Flow cytometry analysis

Briefly, after 21 days and colony formation, the colonies of each group were entirely suspended in the medium. Cell suspensions were then transferred into flow cytometry tubes and centrifuged for 5 minutes at 210-230 g. After centrifugation, the supernatants were discarded, and 1 μl of CD34 antibodies (BioLegend, USA) was added to tubes of each group and placed in a dark environment at 4°C for 30 minutes. Then, the cells were washed three times in phosphate-buffered saline (PBS) Solution (InoClon, Iran) and resuspended in 1 ml PBS solution and examined by flow cytometry instrument (FACSCalibur, BD, USA). In each group, 10,000 cells were analyzed by flow cytometry.

### Karyotype analysis

The karyotype analysis of hematopoietic-like stem cells was performed by a protocol developed by Chou et al. (17).

### Statistical analysis

Each experiment was performed triplicate, and the entire experiment was repeated at three different times. All data that corresponded to the three separate experiments were expressed as means ± SD. The distribution of parameters was evaluated using the Kolmogorov Smirnov test. The independent t test was used to estimate the difference between groups. Statistical analyses were performed using SPSS-18 software. P<0.05 were considered statistically significant.

## Results

### Quantitative real-time polymerase chain reaction analysis of *CD34, CD38,* and *GATA-1* genes

**Fig.1:**
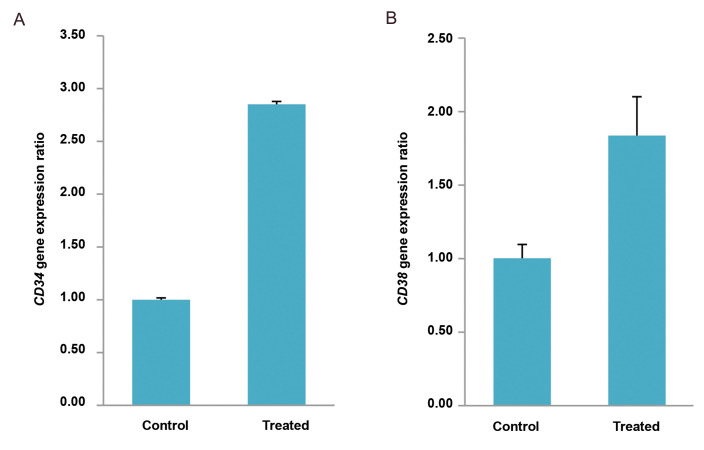
The expression level of hematopoietic-like stem cells genes in the 15 mT group. **A.** The expression level of CD34 gene expression is significantly increased compared to the control group (1.00 ± 0.02 Vs. 6.36 ± 0.08, P<0.001). **B.** The expression level of CD38 gene expression is significantly increased compared to the control group (1.00 ± 0.02 Vs. 3.81 ± 0.30, P<0.005). Data are presented as (mean ± SD).

**Fig.2:**
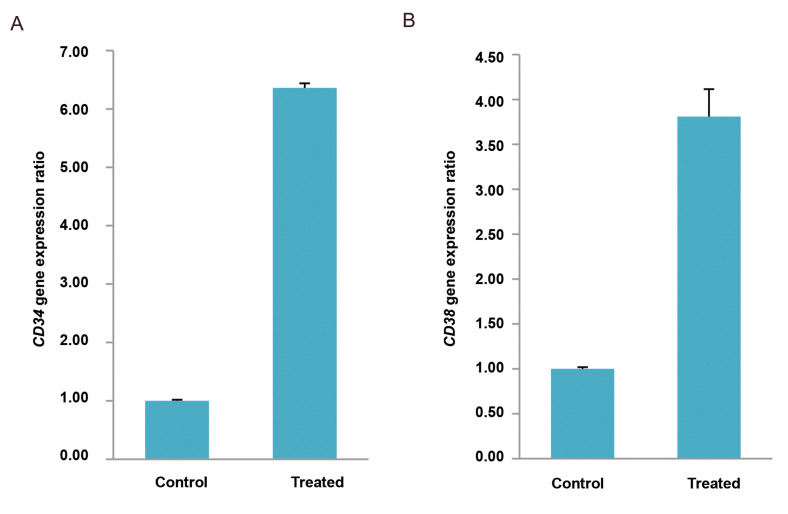
The expression level of hematopoietic-like stem cells genes in the 15 mT group. A. The expression level of CD34 gene expression is significantly increased compared to the control group (1.00 ± 0.02 Vs. 6.36 ± 0.08, P<0.001). B. The expression level of CD38 gene expression is significantly increased compared to the control group (1.00 ± 0.02 Vs. 3.81 ± 0.30, P<0.005). Data are presented as (mean ± SD)

**Fig.3:**
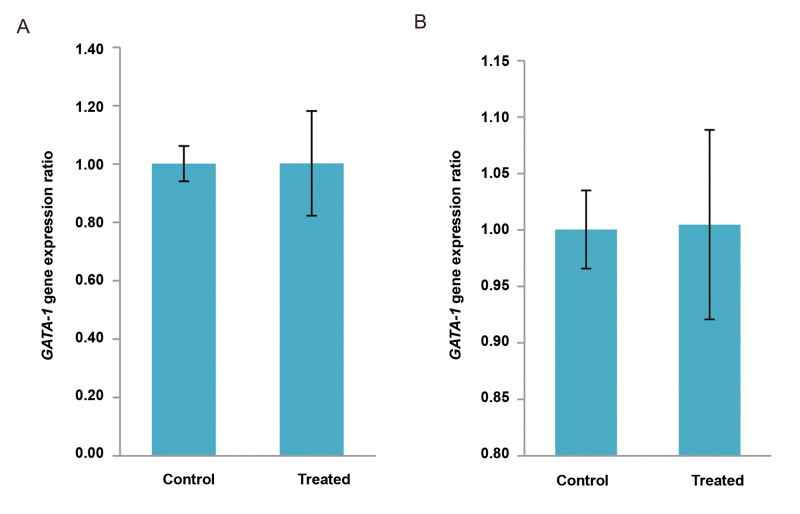
The Expression level of GATA-1 gene in hematopoietic-like stem cells. The expression of the GATA-1 gene did not change compared to the control group. **A.** The expression level of GATA-1 gene in the 10 mT group (1.00 ± 0.06 Vs. 1.00 ± 0.18, P<0.005). **B.** The expression level of GATA-1 gene in the 15 mT group (1.00 ± 0.03 Vs. 1.00 ± 0.08, P<0.005). Data are presented as (mean ± SD).

To determine the differentiation into the hematopoietic lineage using specific *CD34, CD38, *and *GATA-1* genes and quantitative real-time PCR, the change in expression of these genes was analyzed in comparison with the control group. As shown in Figures 1-3, *CD34* and *CD38 *expression in exposed groups show a significant increase compared to the control group (P<0.05). This increase in gene expression levels were 2.85 and 1.84 folds, respectively, in the 10 mT group and 6.36 and 3.81 folds, respectively, in the 15 mT group. Therefore, electromagnetic waves have played a role in the reprogramming of fibroblast cells. The expression of the *GATA-1* gene in exposed groups was not altered in comparison with the control group (P<0.05).

### Flow Cytometry analysis of CD34 Marker

**Fig.4:**
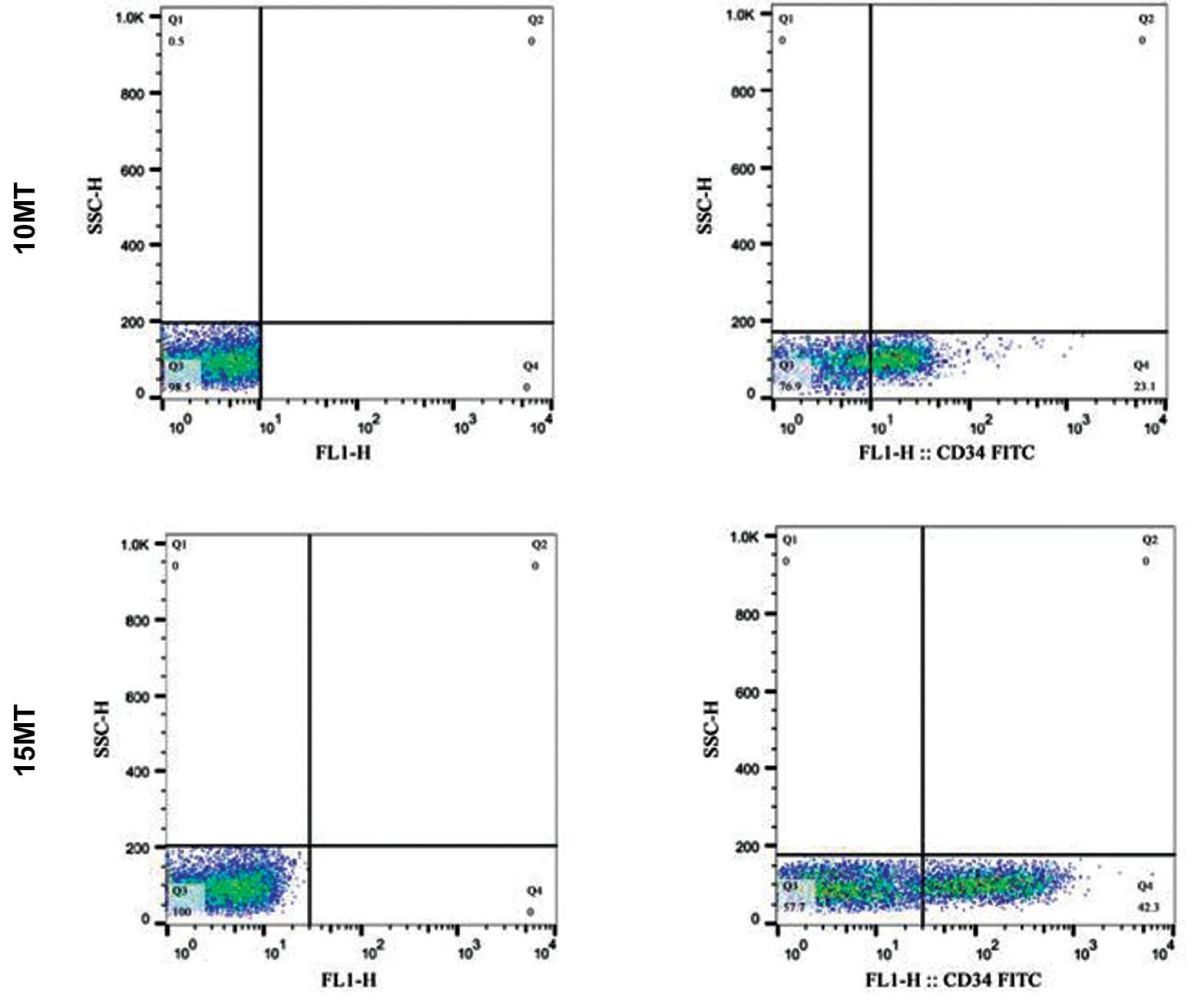
The image shown on the left side is the control sample, and the right model shown the cells treated with the CD34 antibody. The positive regions were adjusted according to the control isotype antibody reaction. (P<0.05). **Upper Image**: Flow cytometric results of CD34 antigen in the 10mT group. A total of 2310 (event) cells express the CD34 marker.** Lower Image**: Flow cytometric results of CD34 antigen in the 15mT group. A total of 4230 (event) cells express the CD34 marker.

Based on flow cytometric results shown in Figure 4, electromagnetic waves have caused the expression of CD34 marker at the surface of reprogrammed cells. This expression is about 42.3% in the 15mT group and 23.1% in the 10 mT group.

### Karyotype analysis

**Fig.5:**
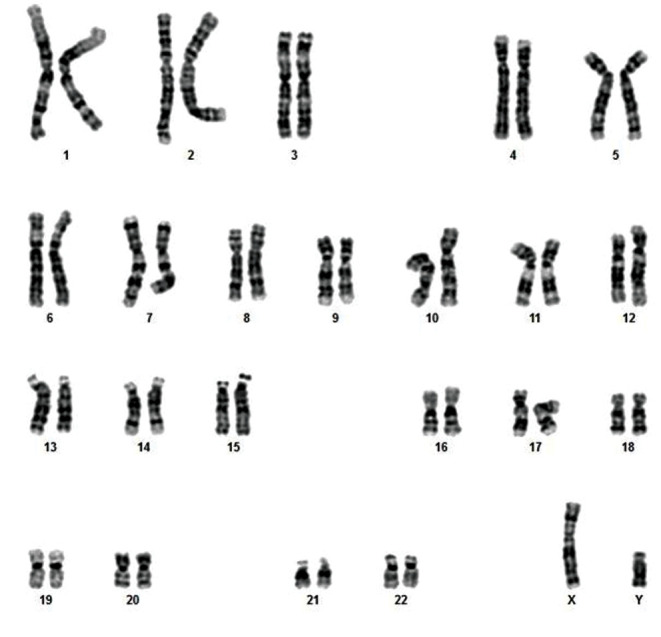
Hematopoietic-like Stem cells shows normal Karyotype.

Based on the karyotype analysis results shown in Figure 5, the karyotype of Hematopoietic-like stem cells shows normal karyotype.

**Table 1:** Genes primer sequence


Gene	Primer sequence (5ˊ-3ˊ)	Amplicon size (bp)

*CD34*	F: CTACAACACCTAGTACCCTTGGA	185
	R: GGTGAACACTGTGCTGATTACA	
*CD38*	F: AGACTGCCAAAGTGTATGGGA	118
	R: GCAAGGTACGGTCTGAGTTCC	
*GATA-1*	F: CTGTCCCCAATAGTGCTTATGG	88
	R: GAATAGGCTGCTGAATTGAGGG	
*HPRT*	F: CCTGGCGTCGTGATTAGTGAT	131
	R: AGACGTTCAGTCCTGTCCATAA	


## Discussion

Electromagnetic waves, as one of the critical forces of nature, are physical energy charged by electrical objects and can influence the movement of other contradictory objects in this field. It was shown in this study that this physical energy can reprogram the cell and affect the fate of the cells. Exposure of cells to electromagnetic waves improves programming in somatic cells (18). Interestingly, exposure to electromagnetic waves with only one Yamanaka’s agent, Oct4, and electromagnetic waves can fill up other factors, such as Sox2, Klf4, and c-Myc to reprogramming (16). These results provided a new window for the efficient production of iPSCs. Therefore, electromagnetic wave base reprogramming can ultimately offer a solution for the efficient and noninvasive cellular planning in regenerative therapies. The current study is a step forward in defining the main factors for inducing pluripotent cells from human fibroblasts.

Similar to ES cells, iPSCs should not be used directly in cell therapy, since the ability to make tumors is one of the inherent characteristics of these cells (9, 19). The ES cells and iPSCs need to be converted into functional cells before used in cell therapy. Recent advances in the direct reprogramming of cells that reprogram a type of somatic cell into a different kind without passing through the pluripotent state is a new solution for the generation of functional cells (20). The first study, in this case, is the expression of a transcription factor (MyoD) in fibroblast cells and the transformation of these cells into myoblasts (21). To date, many functional cells, including neurons, cardiomyocytes, stem cells, neuronal precursors, hepatocytes, and hematopoietic stem cells, have been obtained *in vitro* from fibroblast cells and other somatic cells (22, 23). The flexibility of fibroblasts and the success of their conversion to different types of cells have led to efforts to produce HSC cells from these cells as an alternative strategy for stem cell-based methods.

In the hematopoietic system, the hematopoietic stem cell is only cells that capable of differentiating into all blood cell types and self-renewal. This ability and the ability of these cells to fill the hematopoietic tissue of the individual after receiving the transplant makes use of these cells in regenerative medicine (24). Allogeneic and autologous stem cell transplantation has a disadvantage despite its widespread use in medicine. In an autologous transplant, patients with hematological diseases have the potential to transmit cancer cells to an individual. Allogeneic transplantation also usually results in Graft versus host disease (GVHD) due to minor differences in HLA type between the donor and the recipient (25). Despite medical advances made in HLA typing of individuals, GVHD is the leading cause of death in 60-80% of recipients of transplantation from non-native relatives (26). For this reason, the achievement of hematopoietic stem cells from another type of individual human cells is one of the primary goals of regenerative medicine.

In this study, it was shown that Human primary fibroblast cells could be directly reprogrammed to hematopoieticlike stem cells by exposure to electromagnetic waves and then cultured in a medium containing hematopoietic growth factors. Human primary fibroblast cells appear to be an accessible and safe cell population for cell reprogramming. Reprogramming cells without the use of viral agents is the most crucial goal of this study.

## Conclusion

Because the use of retroviruses has many disadvantages, we are going to reprogram the distinct human fibroblast cells into hematopoietic stem cells by using electromagnetic fields. After exposure of fibroblast cells to electromagnetic fields and then placed in a hematopoietic differentiation medium, the expression of *CD34* and *CD38* genes were measured in 10 and 15 mT groups. The results showed that the expression of these genes after exposure to electromagnetic fields increased. The expression of the CD34 gene in the 10mT and 15mTesla group increased by 2.85 and 6.36 times, respectively, while the expression of the *CD38* gene in the 10 mT group was 1.84 versus 3.81, in the 15 mT group. The expression of the *GATA-1* gene in the 10 mT and 15 mT groups was not significantly different from the control group.

It seems that this method would be suitable for reprogramming the differentiated Human primary fibroblast cells into hematopoietic-like Stem cells and also does not have risks for using retroviruses.

## References

[1] Dulak J, Szade K, Szade A, Nowak W, Józkowicz A (2015). Adult stem cells: hopes and hypes of regenerative medicine. Acta Biochim Pol.

[2] Wernig M, Meissner A, Cassady JP, Jaenisch R (2008). c-Myc is dispensable for direct reprogramming of mouse fibroblasts. Cell Stem Cell.

[3] Okita K, Ichisaka T, Yamanaka S (2007). Generation of germline-competent induced pluripotent stem cells. Nature.

[4] Csobonyeiova M, Polak S, Zamborsky R, Danisovic L (2017). iPS cell technologies and their prospect for bone regeneration and disease modeling: A mini review. J Adv Res.

[5] Eminli S, Foudi A, Stadtfeld M, Maherali N, Ahfeldt T, Mostoslavsky G (2009). Differentiation stage determines potential of hematopoietic cells for reprogramming into induced pluripotent stem cells. Nat Genet.

[6] Zhou W, Freed CR (2009). Adenoviral gene delivery can reprogram human fibroblasts to induced pluripotent stem cells. Stem Cells.

[7] Inoue H, Nagata N, Kurokawa H, Yamanaka S (2014). iPS cells : a game changer for future medicine. EMBO J.

[8] Nienhuis AW, Dunbar CE, Sorrentino BP (2006). Genotoxicity of retroviral integration in hematopoietic cells. Mol Ther.

[9] Aoi T (2016). 10th anniversary of iPS cells: the challenges that lie ahead. J Biochem.

[10] Cho H, Seo YK, Yoon HH, Kim SC, Kim SM, Song KY (2012). Neural stimulation on human bone marrow-derived mesenchymal stem cells by extremely low frequency electromagnetic fields. Biotechnol Prog.

[11] Petri AK, Schmiedchen K, Stunder D, Dechent D, Kraus T, Bailey WH (2017). Biological effects of exposure to static electric fields in humans and vertebrates: a systematic review. Environ Heal.

[12] Sabo J, Mirossay L, Horovcak L, Sarissky M, Mirossay A, Mojzis J (2002). Effects of static magnetic field on human leukemic cell line HL-60. Bioelectrochemistry.

[13] Amaroli A, Chessa MG, Bavestrello G, Bianco B (2013). Effects of an extremely low-frequency electromagnetic field on stress factors: A study in Dictyostelium discoideum cells. Eur J Protistol.

[14] Sun LY, Hsieh DK, Lin PC, Chiu HT, Chiou TW (2010). Pulsed electromagnetic fields accelerate proliferation and osteogenic gene expression in human bone marrow mesenchymal stem cells during osteogenic differentiation. Bioelectromagnetics.

[15] Huangfu D, Osafune K, Maehr R, Guo W, Eijkelenboom A, Chen S (2008). Induction of pluripotent stem cells from primary human fibroblasts with only Oct4 and Sox2. Nat Biotechnol.

[16] Baek S, Quan X, Kim S, Lengner C, Park JK, Kim J (2014). Electromagnetic fields mediate efficient cell reprogramming into a pluripotent state. ACS Nano.

[17] Chou CH, Yang NK, Liu TY, Tai SK, Hsu DSS, Chen YW (2013). Chromosome instability modulated by BMI1- AURKA signaling drives progression in head and neck cancer. Cancer Res.

[18] Ardeshirylajimi A, Soleimani M (2015). Enhanced growth and osteogenic differentiation of induced pluripotent stem cells by extremely low-frequency electromagnetic field. Cell Mol Biol (Noisy-le-grand).

[19] Kurotsu S, Suzuki T, Ieda M (2017). Direct reprogramming, epigenetics, and cardiac regeneration. J Card Fail.

[20] Xu J, Du Y, Deng H (2015). Direct lineage reprogramming: strategies, mechanisms, and applications. Cell Stem Cell.

[21] Davis RL, Weintraub H, Lassar AB (1987). Expression of a single transfected cDNA converts fibroblasts to myoblasts. Cell.

[22] Lee S, Park C, Han JW, Kim JY, Cho K, Kim EJ (2017). Direct reprogramming of human dermal fibroblasts into endothelial cells using ER71/ETV2. Circ Res.

[23] Sandler VM, Lis R, Liu Y, Kedem A, James D, Elemento O (2014). Reprogramming human endothelial cells to haematopoietic cells requires vascular induction. Nature.

[24] Chivu-Economescu M, Rubach M (2016). Hematopoietic stem cells therapies. Curr Stem Cell Res Ther.

[25] Amouzegar A, Dey BR, Spitzer TR (2019). Peripheral Blood or bone marrow stem cells?. Practical Considerations in hematopoietic stem cell transplantation. Transfus Med Rev.

[26] Petersdorf EW (2013). The major histocompatibility complex: a model for understanding graft-versus-host disease. Blood.

